# Quantitative analysis of artificial intelligence on liver cancer: A bibliometric analysis

**DOI:** 10.3389/fonc.2023.990306

**Published:** 2023-02-16

**Authors:** Ming Xiong, Yaona Xu, Yang Zhao, Si He, Qihan Zhu, Yi Wu, Xiaofei Hu, Li Liu

**Affiliations:** ^1^ Department of Digital Medicine, School of Biomedical Engineering and Medical Imaging, Third Military Medical University (Army Medical University), Chongqing, China; ^2^ Department of Nuclear Medicine, Southwest Hospital, Third Military Medical University (Army Medical University), Chongqing, China; ^3^ Department of Ultrasound, Southwest Hospital, Third Military Medical University (Army Medical University), Chongqing, China

**Keywords:** bibliometrics, VOSviewer, Citespace, artificial intelligence, liver cancer

## Abstract

**Objective:**

To provide the current research progress, hotspots, and emerging trends for AI in liver cancer, we have compiled a relative comprehensive and quantitative report on the research of liver disease using artificial intelligence by employing bibliometrics in this study.

**Methods:**

In this study, the Web of Science Core Collection (WoSCC) database was used to perform systematic searches using keywords and a manual screening strategy, VOSviewer was used to analyze the degree of cooperation between countries/regions and institutions, as well as the co-occurrence of cooperation between authors and cited authors. Citespace was applied to generate a dual map to analyze the relationship of citing journals and citied journals and conduct a strong citation bursts ranking analysis of references. Online SRplot was used for in-depth keyword analysis and Microsoft Excel 2019 was used to collect the targeted variables from retrieved articles.

**Results:**

1724 papers were collected in this study, including 1547 original articles and 177 reviews. The study of AI in liver cancer mostly began from 2003 and has developed rapidly from 2017. China has the largest number of publications, and the United States has the highest H-index and total citation counts. The top three most productive institutions are the League of European Research Universities, Sun Yat Sen University, and Zhejiang University. Jasjit S. Suri and *Frontiers in Oncology* are the most published author and journal, respectively. Keyword analysis showed that in addition to the research on liver cancer, research on liver cirrhosis, fatty liver disease, and liver fibrosis were also common. Computed tomography was the most used diagnostic tool, followed by ultrasound and magnetic resonance imaging. The diagnosis and differential diagnosis of liver cancer are currently the most widely adopted research goals, and comprehensive analyses of multi-type data and postoperative analysis of patients with advanced liver cancer are rare. The use of convolutional neural networks is the main technical method used in studies of AI on liver cancer.

**Conclusion:**

AI has undergone rapid development and has a wide application in the diagnosis and treatment of liver diseases, especially in China. Imaging is an indispensable tool in this filed. Mmulti-type data fusion analysis and development of multimodal treatment plans for liver cancer could become the major trend of future research in AI in liver cancer.

## Introduction

Liver cancer is an extremely aggressive malignant tumor, ranking 7^th^ in incidence and 4^th^ in mortality worldwide. Studies have revealed that liver cancer has a high recurrence rate and low recovery rate, especially after the middle and late stages, and the 5-year survival rate of liver cancer is only 5-30% (1, 2). Therefore, it has become a global health problem. Despite advances in diagnosis and treatment of liver cancer, including improved diagnostic imaging accuracy and improved survival after neoadjuvant or conversion therapy, but it is limited. Accurate screening of early-stage liver cancer patients and high-risk patients, and rational treatment decisions for patients with advanced stage liver cancer are of great significance for improving the quality of life of patients.

With the development of medical big data and computer technology, artificial intelligence (AI) based on machine learning and deep learning has been widely used in current medical research (3–6). Through self-learning, summary, and induction of data, it can produce an intelligent reasoning system and choose the optimal solution to guide clinical decision-making (7). Original AI was based on traditional machine-learning methods, including support vector machine and random forest models, which all relied on human experience for learning and simple summary. As early as 2003, Hussain constructed a predictive system consisting of 12 genes, with Fisher’s linear classifier, for predicting early recurrence in patients with hepatocellular carcinoma (HCC) (8). During this period, most studies have focused on simple analyses of data, such as genes and molecules (9–11). With the standardization of imaging diagnosis and its important role in the clinical diagnosis of liver cancer, AI research based on imaging has emerged by extracting high-throughput features that cannot be detected and defined by human eyes from large-scale image data to establish an intelligent decision -making model to assist clinical decision-making (12, 13). In particular, deep learning based on convolutional neural networks (CNNs) has promoted progress in liver cancer research (14–19).

As more and more researchers are interested in the use of AI in liver cancer, a large number of related studies have started being published. For example, reviews describing an overview of deep learning, convolutional neural networks and other AI technologies applications in liver cancer (20–22), reviews on the applications of AI on assisted imaging in diagnosis, prognosis and detection of liver cancer (23–25), and explained the latest research, on limitations and future development trends of AI have all been recently published. However, current reviews may be unable to explore grasp the latest research trends and hotspots in this field because of lack of a large number of publications. Meanwhile, there is a lack of quantitative analysis of all literature in this field. Additionally, a summary and quantitative analyses of the global development trend and research hotspots of AI in liver cancer is of great importance for future research. Bibliometrics is a method of information visualization which can achieve quantitative analysis of literature in a specific research field in a worldwide context through statistical methods and visualizing the results with the help of software (26–29). Bibliometrics plays an important role in sorting out development trends and research hotspots of a given field and has been widely used in many fields (26–29).

Therefore, we aimed to quantitatively analyze existing studies involving AI in liver cancer using bibliometrics to provide the current research progress, hotspots, and emerging trends for AI in liver cancer which may help researchers better understand grasp future research interest. Information was collated regarding countries/regions, institutions, authors, and journals with the highest citations and publications and keywords.

## Methods

### Data sources and search strategies

**Figure 1:**
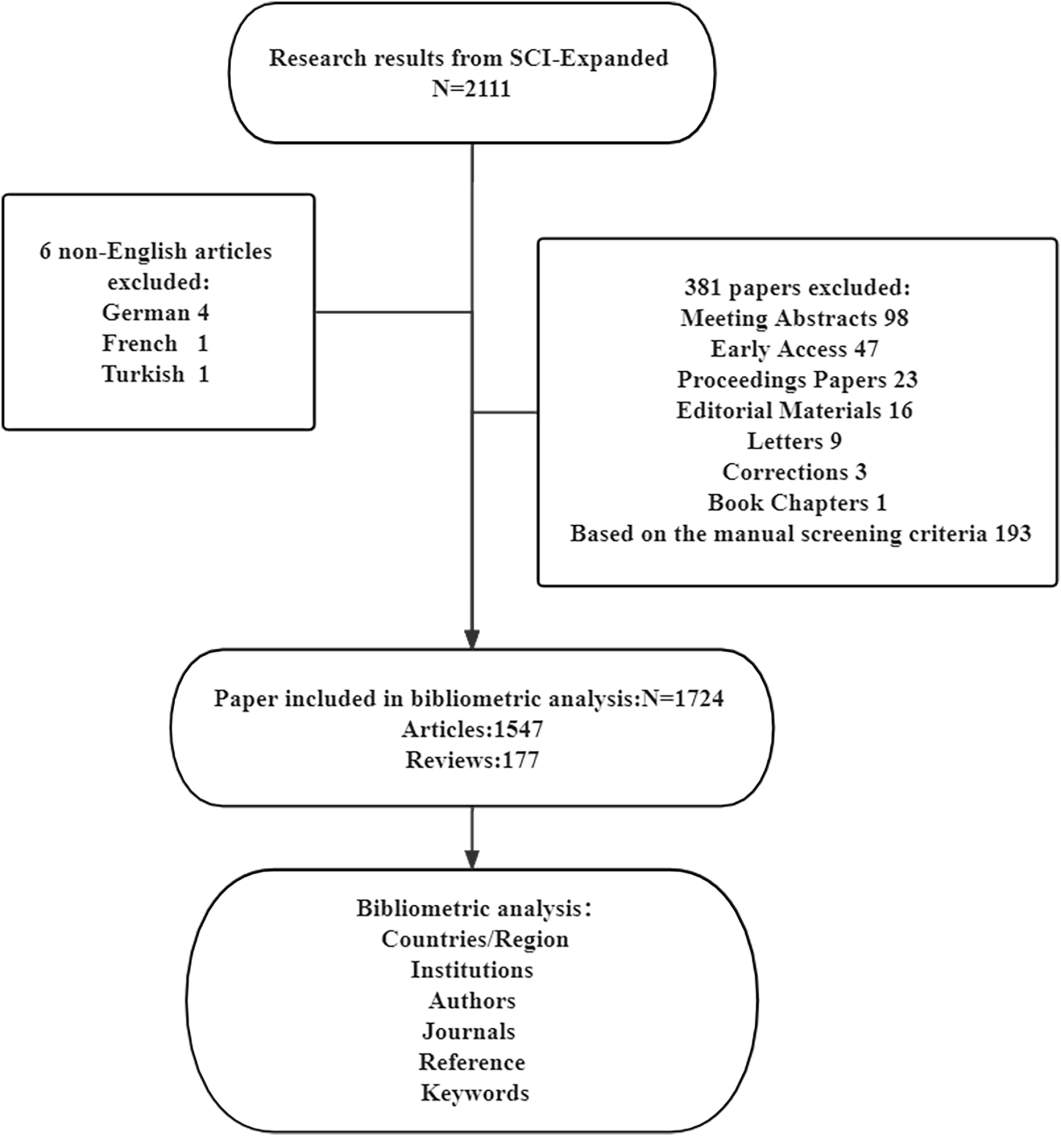
Flowchart of the search process in the study.

The Web of Science Core Collection (WoSCC), which is a standardized and comprehensive dataset, was used to compile the publication dataset in this study. AI is a branch of computer science and a technology that uses machines to simulate human intelligence. AI in this paper mainly includes traditional machine learning and the most popular deep learning algorithms. Therefore, the searching query string was described as follows: TS = (((liver OR hepatic) NEAR/1 (cancer* OR tumor* OR tumor* OR disease OR lesion* OR carcinoma*)) OR “hepatocellular carcinoma” OR “HCC”) AND TS = (((automated OR intelligent) NEAR/1 (classification OR diagnosis OR segment* OR detect*)) OR “artificial intelligence” OR “deep learning” OR “convolutional neural network*” OR “machine learning” OR “CNNs” OR “artificial neural network*” OR “computer-aided” OR “Bayes* network*” OR “supervised learning” OR “unsupervised clustering” OR “computer-assisted” OR (deep NEAR/1 network*) OR “ensemble learning”). The retrieval was carried out on January 18, 2022. **Figure 1** shows the workflow of the retrieval strategy in this research.

### Strategy of manual screening

According to our research area, which focuses on the applications of AI in liver cancer, we designed the following search items: the papers for analysis were restricted to those that (1) were written in English, (2) focused on the field of liver cancer, (3) involved AI technologies. After the preliminary search, 2111 papers were included, and then we conducted further manual screening. In the manual screening process, all papers are divided into relevant, uncertain and excluded categories. Papers marked as unsure were screened by three of the authors (XH, LL, and MX) and discussed to determine whether they should be included. Unlike a systematic review, bibliometric analysis only requires screening of abstracts and full texts are only screened when necessary. According to the screening criteria, 193 papers were excluded because did not focus on the relevant field. Finally, 1724 papers were included in our study.

### Bibliometric analysis and visualization

The analysis of the global trend of publications and citations and productive countries/regions is mainly to comprehensively understand the development trends of AI on liver cancer from beginning to end. The analysis of institutions, authors, and co-cited authors can quantitatively describe the strength of the cooperation between authors and institutions (30–32). Additionally, the analysis of top journals can analyze the level of cooperation and relationships in the concentrated fields of journals, which is beneficial to cross field cooperation in research (32). In particular, cluster co-occurrence analysis of keywords from different perspectives such as disease, data type, clinical goals, and clinical methods can help us understand the main topics and research trends in the current field of AI in liver cancer field.

We used VOSviewer (version 1.6.18) (33) and Citespace (version 6.1.R1) (34, 35) to perform bibliometric visual analysis on the data. VOSviewer was used to analyze the degree of cooperation between countries/regions and institutions, as well as the co-occurrence of cooperation between authors and cited authors. We used Citespace to generate a dual map, which showed the relationship between the main distribution fields of citing journals and citied journals. At the same time, we used Citespace to conduct a strong citation bursts ranking analysis of references. Meanwhile, an online SRplot was used for in-depth keyword analysis. In addition, we used Microsoft Excel 2019 (Microsoft, Redmond, WA) to analyze the targeted files. The top ten of top-cited or productive authors, countries/regions, publications, journals, and institutions were analyzed and tabulated.

In this study, five researchers (M.X., Y.X., Y.Z., S.H., and Q.Z.) were invited to search, download and analyze the publications to assure accuracy of data and repeatability of research.

## Results

### Global trend of publications and citations

**Figure 2:**
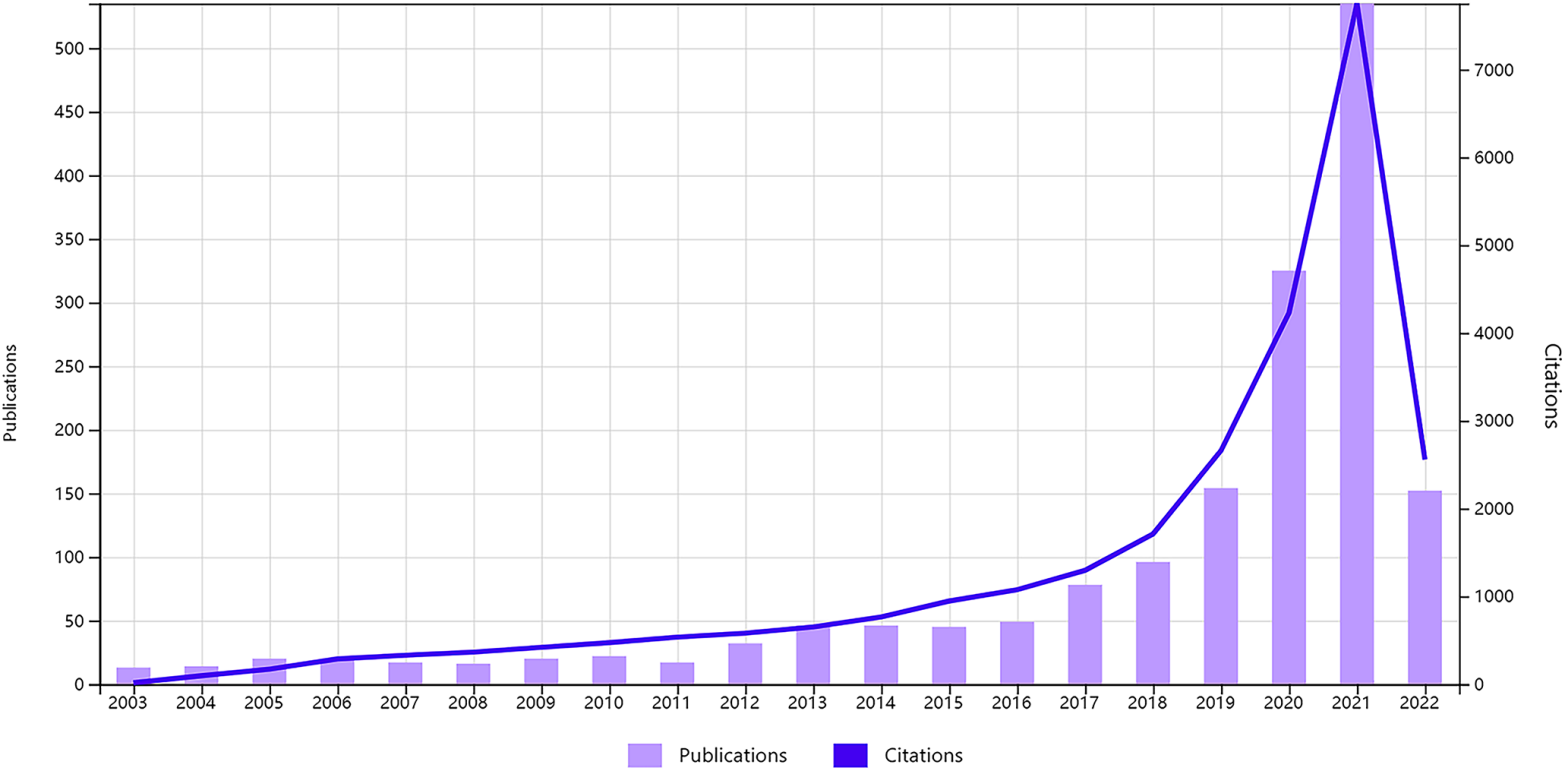
Global trend of publications and citations on artificial intelligence research in liver cancer from 2003 to 2022.

A total of 1724 papers were collected from WoSCC database inception according to our data searching strategy, including 1547 original articles and 177 reviews (**Figure 1**). Research on AI in liver cancer started in 2003 and has increased every year (**Figure 2**). Research has advanced especially rapidly from 2017, accounting for almost 70% of all publications. As of the search date, all papers have been cited 27049 times, and the H-index and average citations per item are 67 and 15.69, respectively. The H-index (36) is a mixed index which could be used as a significant indicator of appraising both the number and level of academic output of a scientific researcher, country, journal, or institution.

### Analysis of productive countries/regions

**Figure 3:**
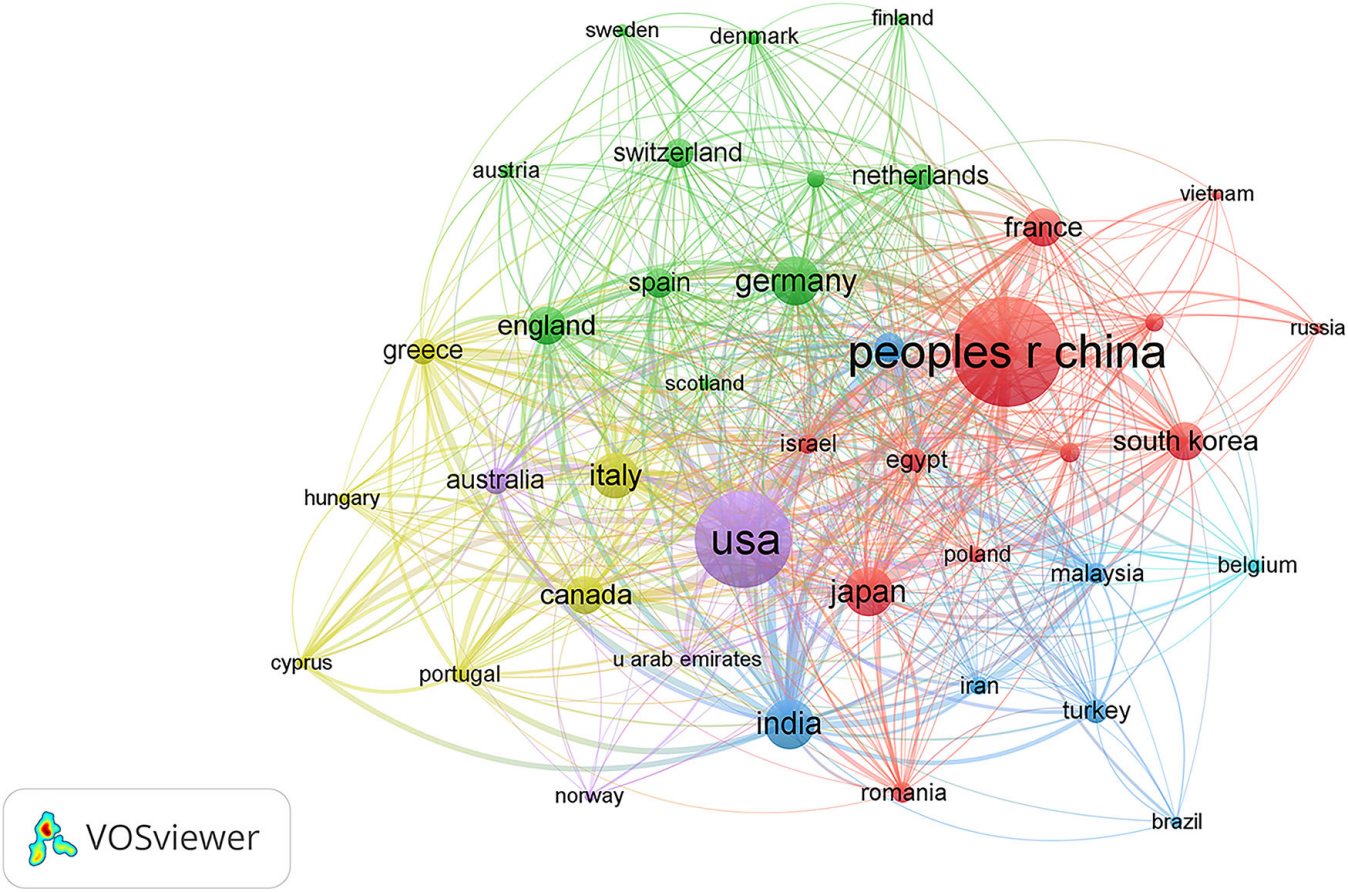
Citation network visualization map of countries/regions. The thickness of the lines reflects the citation strength.

A total of 75 countries/regions had published related articles in this field, of which the top 10 in terms of publication count are China (608), the USA (470), India (129), Germany (122), Japan (118), Italy (105), England (75), South Korea (75), Canada (74), and France (73), accounting for 35.33%, 27.31%, 7.49%, 7.09%, 6.86%, 6.10%, 4.36%, 4.36%, 4.30%, and 4.24% of total publications, respectively (**Table 1**). The USA ranked first in H-index and total citations, with 49 and 10228 citations, respectively, which were both much higher than that of China in second place (38, 7298 citations, respectively). Moreover, the USA was first in terms of average citations per paper, followed by France and Italy. **Figure 3** shows the degree of cooperation between countries when the minimum number of publications was set to at least 5. The lines between nodes indicate co-authorships between countries, where a thicker line indicates stronger cooperation (total link strength [TLS]). The top 5 TLSs were associated with the USA, China, India, Italy, and Canada.

**Table 1:** Top 10 productive countries/regions producing studies related to artificial intelligence in liver cancer.

Rank	Country	Counts	Percentage	H-index	Total citations	Average citation per paper
1	China	608	35.33	38	7298	12
2	USA	470	27.31	49	10228	21.76
3	India	129	7.49	21	1587	12.3
4	Germany	122	7.09	26	2169	17.78
5	Japan	118	6.86	25	2316	19.63
6	Italy	105	6.10	25	2171	20.68
7	England	75	4.36	20	1442	19.23
8	South Korea	75	4.36	15	655	8.73
9	Canada	74	4.30	18	1181	15.96
10	France	73	4.24	24	1541	21.11

### Analysis of productive institutions

**Figure 4:**
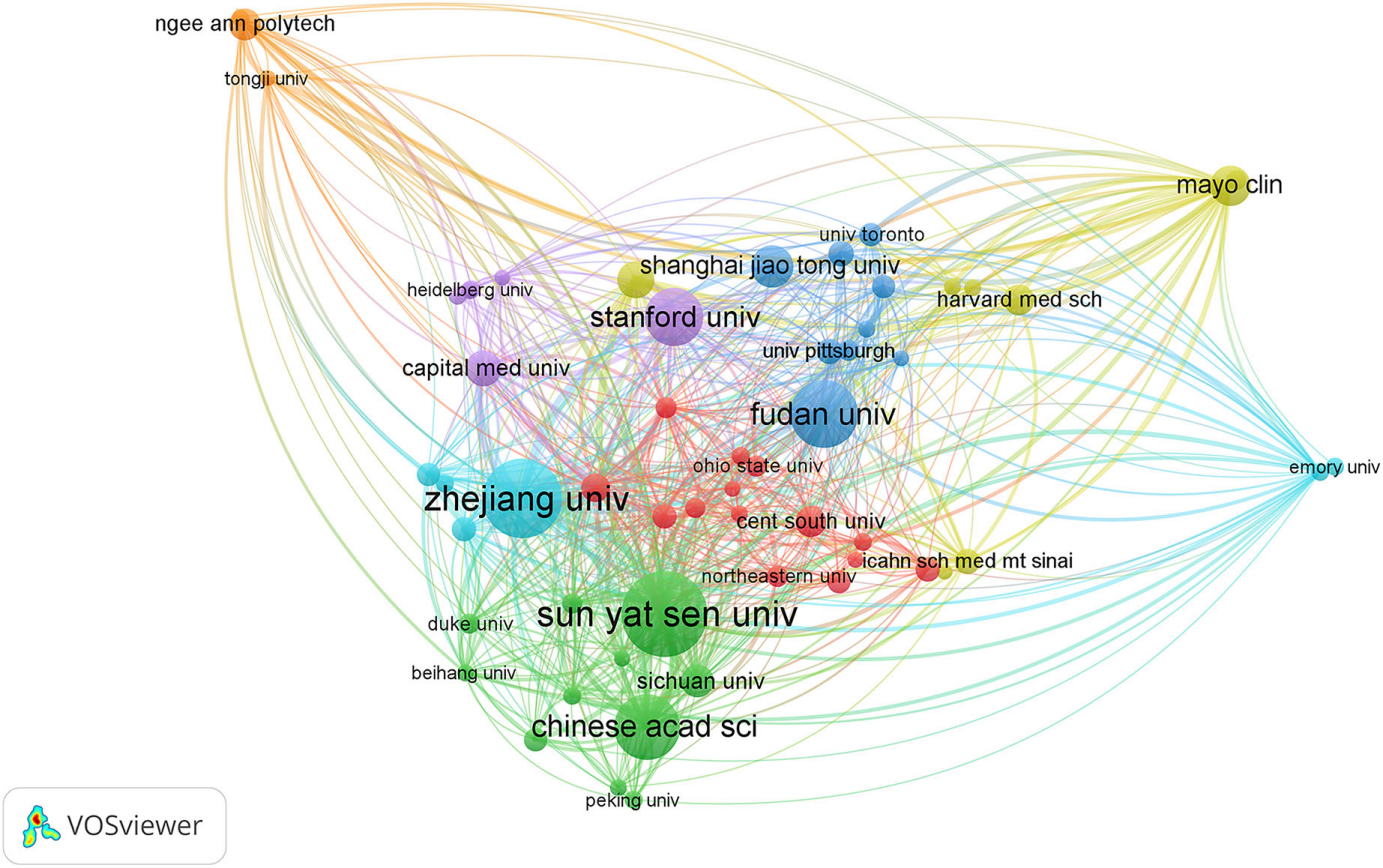
Citation network visualization map of institutions. The thickness of the lines reflects the citation strength.

More than 2000 institutions have participated in research on AI in liver cancer, and the top 10 institutions with the highest contribution are shown in **Table 2**. The top three institutions were the League Of European Research Universities, Sun Yat Sen University, and Zhejiang University with a total of 109, 62, and 58 articles. **Figure 4** shows the TLS between institutions, and the top 3 TLSs were associated with Sun Yat Sen University (TLS = 187), Zhejiang University (TLS = 173), and the Mayo Clinic (TLS = 124).

**Table 2:** Top 10 institutes with publications researching the use of artificial intelligence in liver cancer.

Rank	Institutions	Countries/regions	NP	H-index	NC	Average per item
1	League Of European Research Universities	Belgium	109	25	2746	25.49
2	Sun Yat Sen University	China	62	11	704	11.61
3	Zhejiang University	China	58	10	349	6.19
4	University Of Texas System	USA	57	17	1000	17.93
5	Chinese Academy Of Sciences	China	55	15	799	14.65
6	Fudan University	China	49	10	997	20.45
7	Udice French Research Universities	France	45	20	1103	24.62
8	Harvard University	USA	42	14	766	18.31
9	Stanford University	USA	40	19	791	20.25
10	University Of California System	USA	36	14	795	22.25

NP, number of publications; NC, number of citations.

### Analysis of authors and co-cited authors

**Figure 5:**
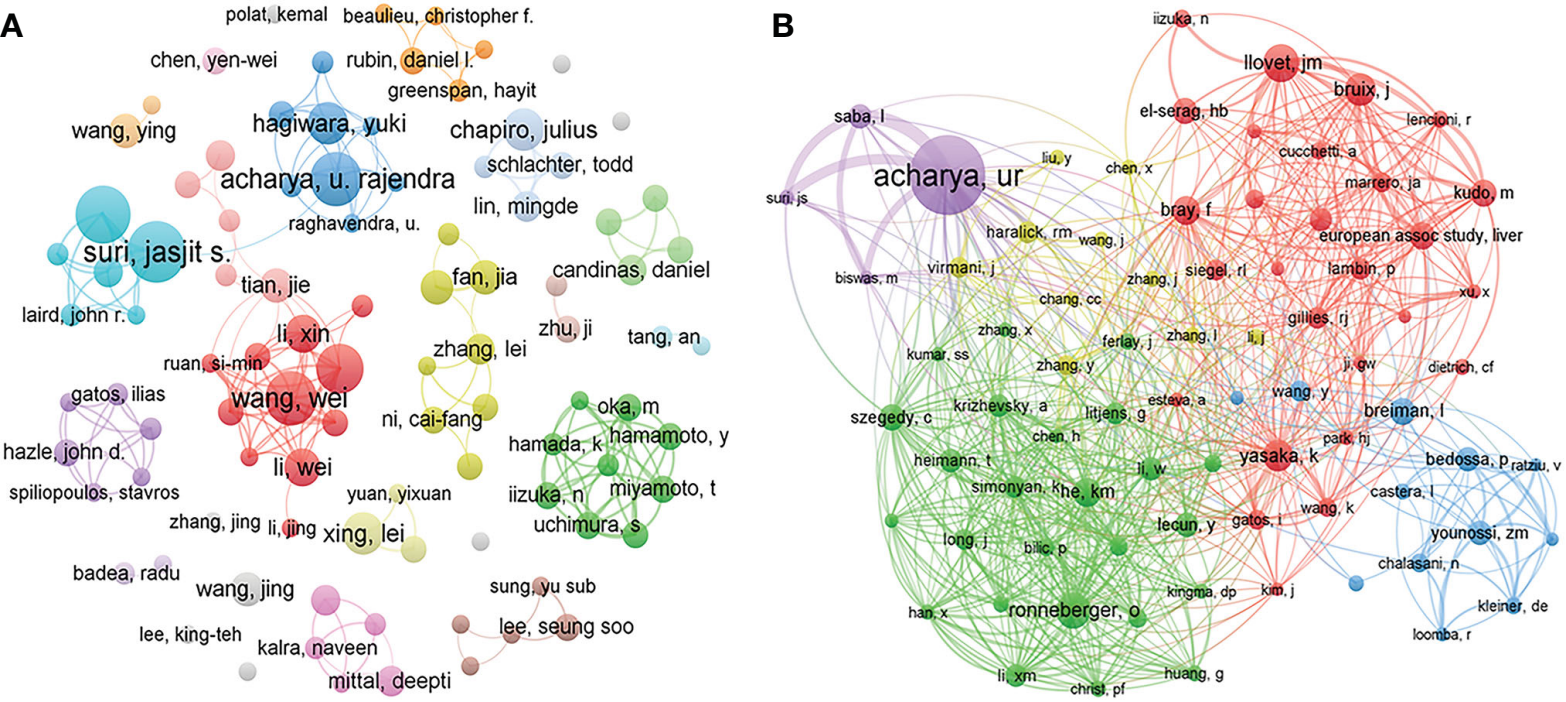
Visualization map of co-authorship **(A)** and co-citation **(B)** analyses of authors. The thickness of the lines reflects the citation strength.

A total of 9916 authors and 37290 co-cited authors were included in the study. **Table 3** shows the top 10 most productive authors, including 4 Chinese, 3 American, 2 Singaporean, and 1 Italian author. Jasjit S. Suri, Luca Saba, and Udyavara Rajendra Acharya were the top 3 authors, with 18, 17, and 15 articles, respectively. VOSviewer was also used to visualize the map of co-authorship of authors and co-citations (**Figure 5**). There were 78 authors with more than 45 citations, among which the top 3 TLSs were associated with U.R. Acharya (TLS = 2274), L. Saba (TLS = 1299), and O. Ronneberger (TLS = 1102).

**Table 3:** The 10 most productive authors of publications researching the use of artificial intelligence in liver cancer.

Rank	Author	Country	Count	Total citations	H-index	Average per item
1	Suri, Jasjit S.	USA	18	456	11	25.33
2	Saba, Luca	Italy	17	371	10	21.82
3	Acharya, Udyavara Rajendra	ingapore	15	519	11	34.6
4	Kuang, Ming	China	14	214	6	15.29
5	Chapiro, Julius	USA	12	237	6	19.75
6	Xing, Lei	USA	11	318	7	28.91
7	Hagiwara, Yuki	Singapore	11	207	6	18.82
8	Tian, Jie	China	11	191	7	17.36
9	Wang, Wei	China	11	177	5	16.09
10	Fan, Jiahao	China	9	56	5	6.22

### Analysis of top journals

**Figure 6:**
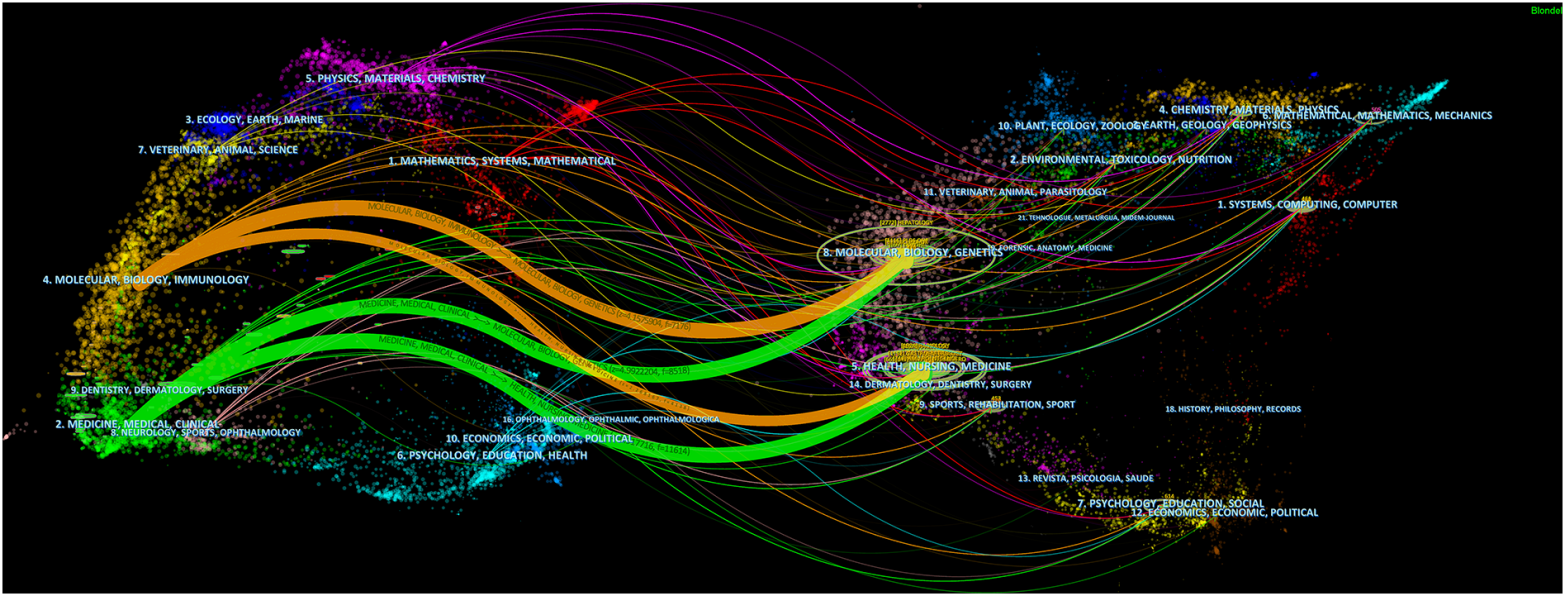
A dual-map overlap of journals with studies researching artificial intelligence in liver cancer.

All related studies have been published in 585 journals. **Table 4** shows the top 10 most productive journals, including their paper count, impact factor (IF), Journal Citation Ranking (JCR), H-index, and total count. The top 3 journals were *Frontiers in Oncology* (50, 2.90%), *European Radiology* (45, 2.61%), and *Scientific Reports* (41, 2.38%). Of the top 10 journals, there are 4 comprehensive medical journals (*Frontiers in Oncology*, *Scientific Reports*, *PLoS One*, *International Journal of Computer Assisted Radiology and Surgery*), 1 hepatobiliary professional journal (*World Journal of Gastroenterology*), 1 medical imaging journal (*European Radiology*), and 2 engineering journals (*Medical Physics*, *IEEE Access*). Forty percent of journals had a JCR of Q1. **Figure 6** shows the dual map of journals and the relationship between citing journals and cited journals. There were mainly four citation paths, and the citing papers were mainly concentrated in three fields: (1) molecular, biology, and immunology; (2) medicine, medical, clinical; and (3) neurology, sports, ophthalmology. The cited papers were mainly located in 3 fields: (1) molecular, biology, genetics; (2) health, nursing, medicine; and (3) dermatology, dentistry, surgery.

**Table 4:** Top 10 journals related to research on artificial intelligence in liver cancer.

Rank	Journal	Count	IF (2020)	JCR (2020)	H-index	Total citations
1	*Frontiers in Oncology*	50	6.244	Q2	8	236
2	*European Radiology*	45	5.315	Q1	16	840
3	*Scientific Reports*	41	4.38	Q1	12	539
4	*Medical Physics*	35	4.071	Q1	13	549
5	*PLoS One*	35	3.24	Q2	15	675
6	*World Journal of Gastroenterology*	31	5.742	Q2	10	286
7	*International Journal of Computer Assisted Radiology and Surgery*	26	2.924	Q2/Q3	11	312
8	*Cancers*	23	6.639	Q1	5	74
9	*Computers in Biology and Medicine*	23	4.589	Q1/Q2	11	370
10	*IEEE Access*	22	3.367	Q2	7	139

IF, impact factor; JCR, Journal Citation Ranking.

### Analysis of top cited references and co-citation references

**Figure 7:**
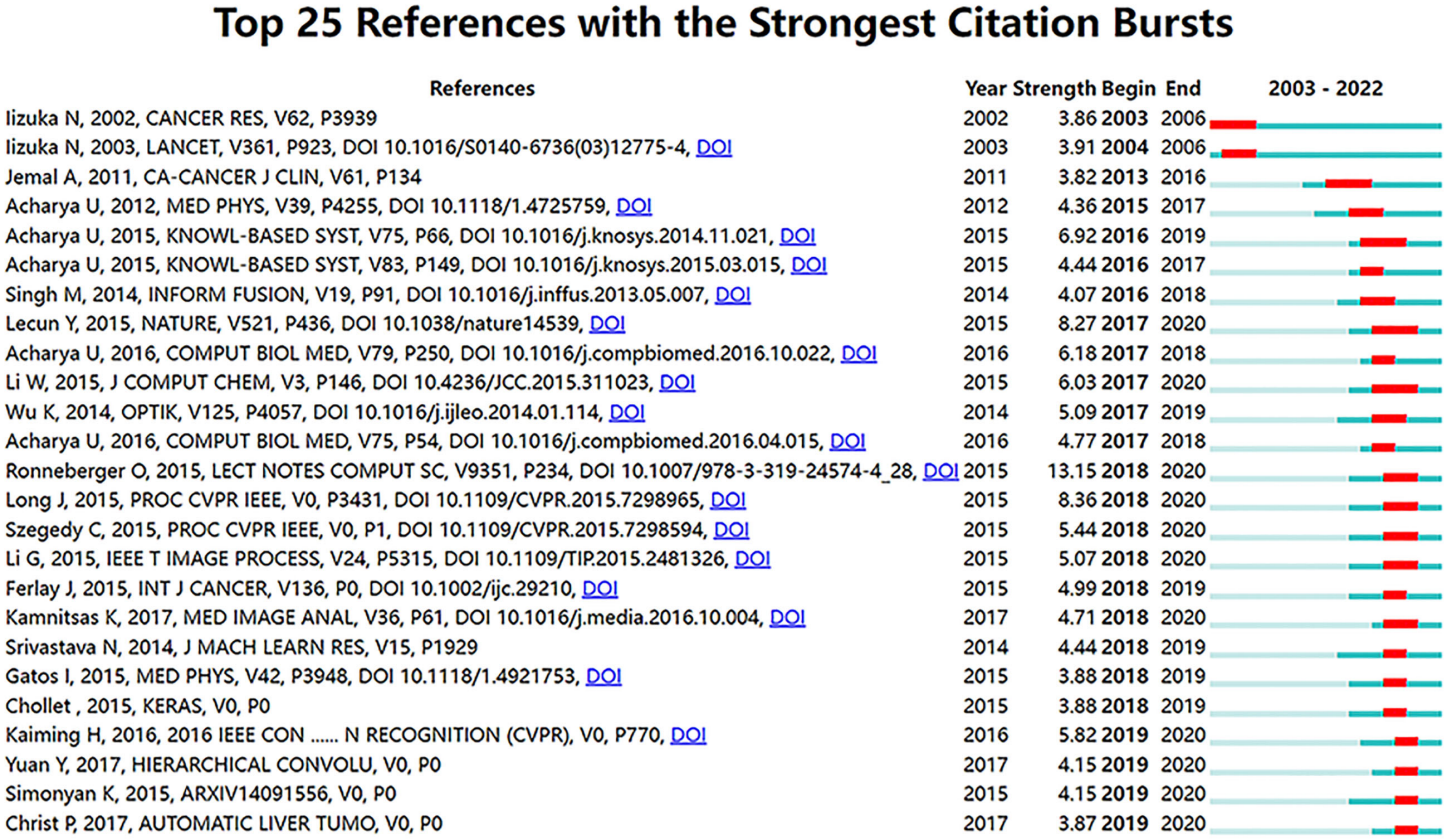
Visualization map of top 25 references with the strongest citation bursts from 2003 to 2022.

**Figure 7** shows the top 25 references with the strongest citation bursts. The explosion of citations in this field began in 2003, and a large number of co-citation references were focused on the period from 2015 to 2019, indicating that research in this field was a hotspot in these years.

### Analysis of keywords

**Figure 8:**
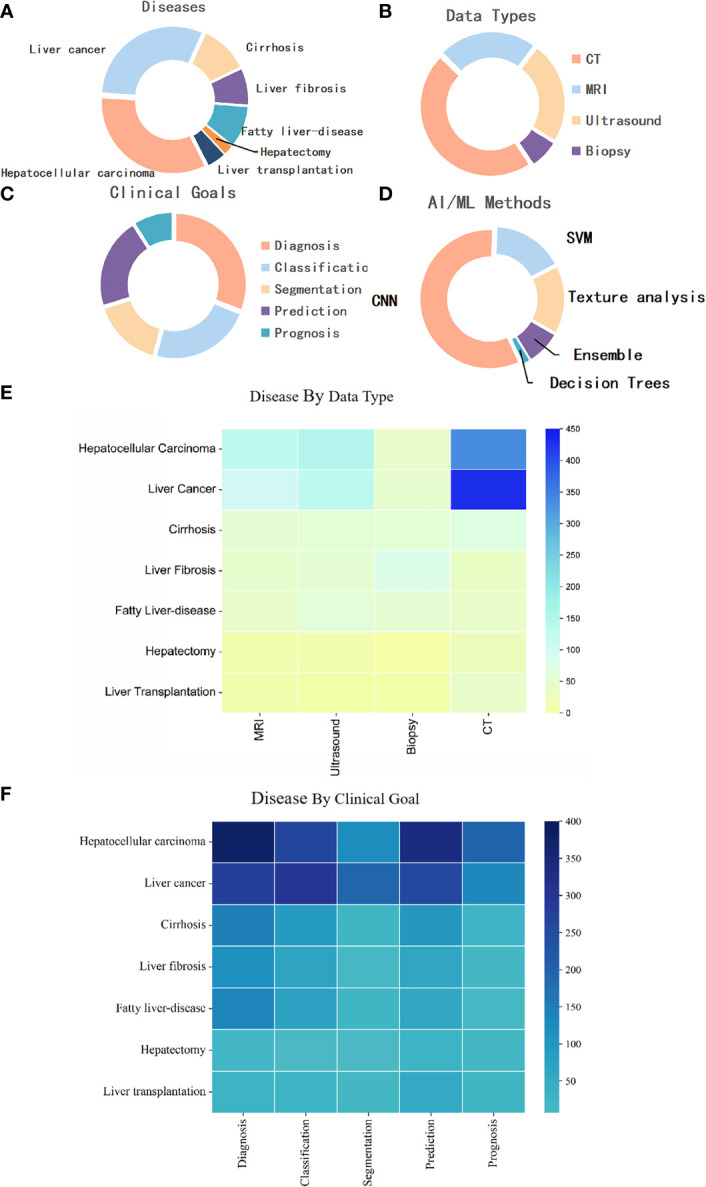
**(A)** Distribution of publications by disease category. **(B)** Distribution of publications by data modality. **(C)** Distribution of publications by goal. **(D)** Distribution of publications by AI/ML method. **(E)** Distribution of publications by disease by data type. **(F)** Distribution of publications by disease by clinical goal. MRI, magnetic resonance imaging; CNN, Convolutional Neural Network; SVM, support vector machine; CT, computed tomography.

An in-depth analysis of keywords from the diseases, data types, clinical goals, and methods (**Figure 8**) were conducted. Most articles focused on liver cancer, and HCC was widely studied as a single type of disease, followed by cirrhosis, fatty liver disease, liver fibrosis, liver transplantation, and hepatectomy, accounting for 30.76%, 33.52%, 11.10%, 9.45%, 8.39%, 4.41%, and 2.37%, respectively (**Figure 8A**). In terms of the data type, computed tomography (CT, 46.79%) was the most used, followed by ultrasound (23.58%), magnetic resonance imaging (MRI, 22.83%), and biopsy (6.79%) (**Figure 8B**). **Figure 8E** shows that in the study of liver cancer, including HCC, CT was the most used, followed by ultrasound and MRI. In addition, CT was mainly used for the research of liver fibrosis, ultrasound was mainly used for the research of fatty liver disease, and biopsy was mainly used for liver fibrosis research (**Figure 8E**). The differential diagnosis of HCC, are the key points, followed by the diagnosis of liver cirrhosis, liver fibrosis and fatty liver disease, are key points among the specific diagnosis, classification, and treatment of liver diseases. In terms of the prognosis of liver disease, the prognosis of HCC is a key focus, and the surgical methods for its treatment mainly include radiofrequency ablation and transarterial chemoembolization (**Figure 8F**). Three quarters of these papers were about diagnosis, classification, segmentation, or prediction, with relatively less attention to prognosis. Moreover, most liver cancer studies used CNNs, with a minority exclusively using more traditional techniques like support vector machine and decision trees (**Figure 8D**).

## Discussion

In this quantitative study, in order to systematically and quantitatively analyze the research status of AI in liver cancer, and explore the future research trends and hotspots in this field, we used a bibliometrics method to analyze the current research status of AI in liver cancer in terms of publication and citation trends, countries/regions and institutions, authors and co-cited authors, journals, cited references and co-citation references, and keywords. Ultimately, 1724 articles focusing on AI in liver cancer were collected from the WoSCC database and analyzed.

Research on AI in liver cancer mainly started in 2003 and entered a stage of rapid development in 2017. China is the most productive country with 35.33% of total publications; however, the USA ranked first according to the H-index, citations, and average citations per paper. It is notable that China, as a country with a high incidence of liver cancer, has a high number of studies on AI in liver cancer. However, most studies in China have limited impact, which may need further improvement from topic selection and research implementation. The League Of European Research Universities is the most productive institution, followed by Sun Yat Sen University and Zhejiang University. This is consistent with the conclusion of the most productive country above. We also found that cooperation between medical and industrial universities contributes to better research. For example, Sun Yat Sen University and the Chinese Academy Of Sciences (the second and fifth most productive institutions, respectively) have a strong TLS. Fu Dan University and Shanghai Jiaotong University also have a strong TLS. This indicated that the combination of medicine with engineering is helpful for the development of AI in medicine. It also suggested that studies should pay attention to the reasonable allocation of research teams.

The top three most productive journals had JCR scores of at least Q2. This shows that the field of AI in liver cancer is relatively mature and has a high level of concern and recognition. Moreover, most of the top 10 journals in this field are medical journals and include a small number of engineering journals, showing that the medical field pays more attention to AI in liver cancer. This suggests that we can consider and design studies from both medical and engineering aspects when conducting research, especially in medicine.

In the in-depth analysis of keywords, we found that most studies focused on liver cancer, especially HCC, showing that this is a research priority of liver disease. The second most common area of research was chronic hepatitis diseases such as liver cirrhosis, liver fibrosis, and fatty liver disease, which are more important for the prevention of liver cancer.

Regarding data type, studies of AI in liver cancer started from the simple data modeling of genetic or molecular data (9–11). With the development of medical imaging, research on medical imaging has been gradually increasing. CT, ultrasound, and MRI are the top three most used data types. First, this may because CT and MRI can be used as the basis for clinical treatment strategies for patients with liver cancer based on guidelines for liver cancer diagnosis. Moreover, ultrasound, as a screening method for patients at high risk of liver cancer, needs to be checked every six months. Therefore, the data volume of these three imaging methods has greatly increased, which has promoted the development of AI in liver cancer (37–39). Second, compared with MRI, CT has the advantages of fast inspection speed and cost-effectiveness, and is an indispensable and important imaging method in the diagnosis and treatment of liver cancer. Finally, although ultrasound is widely used in clinical practice, its image acquisition is seriously affected by the doctor’s operation technique and machine model, the resolution is low, and the processing is difficult. Therefore, it is used less often than CT. However, it is worth noting that contrast-enhanced ultrasound has now been included as a recommended imaging modality for the diagnosis of liver cancer (40, 41) and is also widely used in the development and prognostic evaluation of ultrasound-guided radiofrequency ablation. This suggests that we could pay attention to the important role of ultrasound in liver cancer clinics in future research. At the same time, few studies used pathological, genetic, and other clinical data (42–44). The main reason may be that the medical cost of genetic examination is high and the realization of AI in multiomics research is difficult.

In the cross-analysis of data types and diseases, we found that biopsy was used as an important data type in studies of AI in liver fibrosis. This is mainly because the histopathological examination of liver biopsy is still the gold standard for the diagnosis of liver fibrosis (45). Conventional CT/MRI examinations can observe morphological changes of the liver; however, quantitative assessment of early-stage liver fibrosis is still difficult and is therefore less used. Although ultrasound elastography and magnetic resonance elastography (MRE) are highly effective non-invasive assessment methods in the diagnosis of liver fibrosis, a unified MRE liver elasticity value for liver fibrosis with different etiologies has not been established (46–48). This also indicates that the use of AI to quantitatively analyze liver fibrosis by imaging is a problem worthy of further study. In studies of AI in fatty liver disease, ultrasound is the first choice, mainly because of its high sensitivity in the diagnosis of diffuse fatty liver, convenience, cost-effectiveness, and safety, and plays an important role in judging the status of liver parenchyma.

In terms of clinical goals, the diagnosis and differential diagnosis of liver cancer on medical imaging are still major research priorities (19, 49–53). However, the clinical diagnosis of liver cancer is a comprehensive process, especially because of the variety and atypical characteristics of focal liver lesions. For example, dysplastic nodules in the state of liver cirrhosis have strong malignant potential, especially high-grade dysplastic nodules, and they are difficult to distinguish from early liver cancer in imaging. A comprehensive evaluation of the clinical indicators of the patient is usually required, including alpha-fetoprotein and abnormal prothrombin (54–58). However, there are still few studies that combine multiple types of data such as genetic data, molecular data, imaging data, and clinical indicators, and lack the support of large data and multi-center studies.

Studies on the treatment and prognosis of liver cancer mainly focused on the survival of a specific surgical method (59–66), such as radiofrequency ablation, transarterial chemoembolization and etc. Reports have proven that the modern therapies integrate a variety of neoadjuvant and adjuvant strategies have achieved dramatic improvements in survival, especially for patients with advanced HCC (66, 67). But the division of the patient population, the choice of potentially disclosing novel biomarkers still are controversies and the decision-making of precision treatment methods adapted to the specific patients, AI can play a role in this, but related research has not yet been seen.

In terms of methods used, some studies used traditional algorithms (51–53), such as support vector machine and random forests models, which were mainly concentrated in the early research stage. Since 2012, deep learning with CNNs has been widely used in the field, involving common tasks in the field of machine learning such as diagnosis, prediction, and segmentation, and achieved good results (9, 49, 50, 68–70). Most tumor segmentation tasks use a U-Net method with a good effect, showing that U-Net has a good effect in the segmentation task in the medical field, especially for tasks with small amount of medical data (71, 72). It is estimated that the main research methods in later stages of research are still concentrated in the field of deep learning, which also indicates that future research aims to achieve better results and has higher technical requirements, especially for fusion modeling of multimodal data.

Previous meta-analyses and literature reviews focused on the applications of specific technologies in liver cancer or the development status of specific liver disease (22–29), such as reviewing studies on AI on assisted imaging in the diagnosis, prognosis and detection of liver cancer, or explaining the latest research, limitations, and future development trends of AI in a certain direction. However, they lack a quantitative analysis based on the available literatures. Therefore, a bibliometrics analysis was conducted in our study to summary the research status of AI in liver cancer. Bibliometrics analysis uses mathematical and statistical methods to study the literature system and bibliometric characteristics in a given field to mine the distribution structure, quantitative relationships, and changes of literature in this field. Visual display with the help of special software plays an important role in understanding the current development status and development trend of the field. However, our research also has limitations. First, we only included English articles in the WoSCC database and did not include articles in other databases or languages, which could lead to the omission of many studies. Second, keyword screening may not be perfect and could lead to omission of literature.

## Conclusion

This study used bibliometrics to conduct an in-depth analysis of the published literature on AI in liver cancer. The results showed that AI has undergone rapid development and has a wide application in the diagnosis and treatment of liver diseases, especially in China, which has one of the highest incidences of liver cancer compared to other countries the world. In addition, intelligent analysis of imaging data is the hotspot and focus of current research in this field. However, combined with the current clinical difficulties such as accurate screening of early-stage liver cancer patients and high-risk patients, and selection of reasonable treatment decisions for advanced liver cancer patients, the use of AI for the fusion analysis of multiple types data in the process of diagnosis and treatment of liver cancer and multi-modal treatment decision-making for liver cancer are still relatively rare, and may become a future research trend.

## Data availability statement

The raw data supporting the conclusions of this article will be made available by the authors, without undue reservation.

## Author contributions

LL, XH, and YW conceived the study. MX, YX, YZ, SH, and QZ collected and analyzed the data. MX, YX, XH, and LL wrote the manuscript. LL, XH, and YW revised and reviewed the manuscript. All authors contributed to the article and approved the submitted version.
